# The Effect of Sterilization Methods on the Mechanical Properties of 3D-Printed and Conventional PMMA Materials for Denture Bases of Immediate Obturators

**DOI:** 10.3390/polym17091279

**Published:** 2025-05-07

**Authors:** Anna Cybulska, Katarzyna Mańka-Malara, Michał Krasowski, Jerzy Sokołowski, Jakub Zwoliński, Andrzej Rafalski, Jolanta Kostrzewa-Janicka

**Affiliations:** 1 Department of Prosthodontics, Medical University of Warsaw, 02-091 Warsaw, Poland; 2 Department of Orthodontics, Medical University of Warsaw, 02-091 Warsaw, Poland; 3 University Laboratory of Materials Research, Medical University of Lodz, 90-647 Lodz, Poland; 4 Department of General Dentistry, Medical University of Lodz, 90-647 Lodz, Poland; 5 Department of Head and Neck Oncology, Maria Sklodowska-Curie National Research Institute of Oncology, 02-781 Warsaw, Poland; 6 Station for Radiation Sterilization of Medical Devices and Transplants, Institute of Chemistry and Nuclear Technology, 03-195 Warsaw, Poland

**Keywords:** additive manufacturing, radiation sterilization, pressurized steam sterilization, ethylene oxide sterilization, dentistry, prosthodontics, dental materials, dental prostheses

## Abstract

The use of 3D printing in the fabrication of immediate prosthetic restorations requires the possibility of sterilization. This study aimed to evaluate the effects of different sterilization methods on the parameters of 3D-printing materials for dental prosthesis plates compared to conventional acrylic material. Forty-four samples were prepared for each tested material: Denture 3D+ (NextDent, The Netherlands), Denturetec (Saremco, Switzerland), Optiprint Laviva (Dentona, Germany), and Rapid Simplified (Vertex Dental, Netherlands). The impact strength of the samples was tested in a HIT 5.5P instrument (Zwick Roell, Germany) after three sterilization methods (pressurized steam, ethylene oxide, and radiation) and without sterilization as a control group. Significantly higher energy and impact strength were recorded for the conventional acrylic material. For Nextdent material, the recommended method of sterilization in terms of impaction is autoclave or ethylene oxide sterilization, Saremco—ethylene oxide sterilization, and Denton—ethylene oxide or radiation sterilization. Conventional acrylic material has a higher impact strength than 3D-printed material, which may encourage the selection of this material for restorations requiring higher fracture strength. The possibility of sterilizing the Nextdent 3D-printed material in the autoclave without worsening its durability makes it a recommended choice for digital clinical practice.

## 1. Introduction

Polymeric materials are widely used in dentistry. The dynamic development of digital technologies allows their use during the fabrication of all types of prosthetic restorations. Nowadays, 3D printing is used to produce dental models, occlusal splints, fixed and removable prosthetic restorations, as well as surgical templates and immediate dentures inserted into the foundation immediately after the surgery [1,2,3]. The use of 3D printing makes it possible to precisely produce an object of any shape, which is advantageous when fabricating the removable restoration for a patient after a surgical procedure, especially when the project requires designing the closed hollow obturators after jaw resection due to cancer [4,5,6]. The device, especially the one that has a direct contact with a wound created during the surgery, must be sterile [7,8,9,10,11,12,13].

Sterilization is defined as the process of complete elimination of all living microorganisms (both vegetative and spore forms), using physical, chemical, and mechanical methods [7,9,14,15]. The most-used sterilization methods are pressurized steam in the autoclave, ethylene oxide, and radiation sterilization [7,8,11,16,17]. The immediate denture for patients after maxillectomy has a complicated construction—the hollowed, obturator part may be a challenge for sterilization. In dental offices, the restorations may be sterilized before being given to the patients using autoclaves. Pressurized steam sterilization is a fast, non-toxic, environmentally friendly, and economical process, and thus it is most widely used [9,14,16,18,19]. Sterilized objects are exposed to steam at the appropriate temperature and pressure for a specified period. Most often, the process lasts 15–30 min at 121 °C or 3–4 min at 134 °C with a pressure of 1–2 bars [14,15,16,19]. However, this type of sterilization cannot be used for materials which are sensitive to heat, pressure, or steam [9,13,14,15,16]. In hospitals, the ethylene oxide (EtO) sterilization method can be applied as a low-temperature sterilizing agent for medical products [15,20]. The effectiveness of ethylene oxide sterilization is influenced by gas concentration (450–1200 mg/L), temperature (29–65 °C), humidity (45–85%), and exposure time (1–5 h) [14,20,21]. This process requires special caution and aeration of devices because this gas is toxic, explosive and cancerogenic [8,9,14,16,21,22]. However, the high penetration capacity is advantageous when considering sterilizing appliances or instruments of complicated structure, and it is compatible with most materials used in medicine. For materials that are heat-sensitive, radiation sterilization can be also applied [15,23,24,25,26,27]. The use of radiation techniques, referred to as cold sterilization, use the process of transferring the energy of an electron beam. It is broadly used for polymeric, disposable medical devices, which are often heat-sensitive [5,23]. The gamma rays (γ) of radioactive isotopes and accelerated electron beams (Elektron Beam—EB) are most used [9,22,23,25]. The choice of γ-rays or fast electrons for sterilization is based mainly on the uniformity factor dose requirements. Electron beam technology is preferred for sterilization of small packages of products with limited density due to its speed and the efficiency of the process. For sterilization of products with high and inhomogeneous density, γ-rays are used to achieve the desired homogeneity [9,23,25]. The most frequent doses used for sterilization are 15 kGy and 25 kGy [28]. Despite the high cost of this method, its high reproducibility, lack of overheating, and reduced environmental effects are important [9,25,26,27,28,29]. The consequences of radiation on the polymer vary widely and depend on the structure of the chain, the size of the absorbed dose and the radiation conditions [9,12,22,28,30,31,32]. Additionally, radiation sterilization may cause a change in the polymer resin color [12,15,32]. However, the advantages of radiation sterilization include the ability to sterilize products in a short period of time, reduced environmental risks, the possibility of using any temperature, and that the entire volume of material for unit and bulk packaging may be sterilized simultaneously, thanks to the ability of ionizing radiation to penetrate the packaging and materials. Table 1 contains the comparison of sterilization methods that may be used for medical appliances.

Before choosing a sterilization method for a given medical device, it is necessary to determine its effect on the specific properties of a given product, especially its mechanical properties [15]. There are studies in the literature on the effect of different sterilization methods on medical materials containing polymethylmethacrylate (PMMA), but there are no studies on the effect of different sterilization methods on 3D-printed materials for dentures. When introducing new materials into daily practice, many aspects of their characteristics should be considered. The prosthetic restoration must be durable, especially constructions dedicated for immediate application. There are many studies on the positive effects of tissue healing using immediate dentures; however, the 3D-printed ones are still not broadly tested. The denture for the patient must be safe—both in terms of microbiological cleanness and for the risk for potential breakage. The aim of the study was to evaluate whether the application of different sterilization methods deteriorates the mechanical properties of denture materials for 3D printing. The zero hypothesis of the study was that there would not be significant differences between evaluated groups.

## 2. Materials and Methods

**Figure 1:**
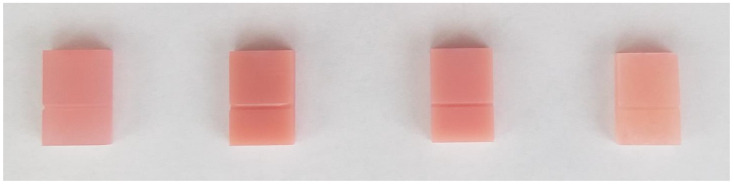
Samples prepared using evaluated materials—from the left side: Denture 3D+, NextDent; Denturetec, Saremco; Optiprint Laviva, Dentona; Rapid Simplified, Vertex Dental.

Forty-four samples were prepared using each of the tested materials for DLP (digital light processing) 3D printing: NextDent Denture 3D+ (NextDent, Soesterberg, The Netherlands), Denturetec (Saremco, Rebstein, Switzerland), Optiprint Laviva (Dentona, Dortmund, Germany), and Rapid Simplified conventional denture fabrication material (Vertex Dental, Soesterberg, Netherlands), which was included in the study as a material for control samples. Materials included in the presented study were biocompatible, dedicated for intraoral application and fabrication of denture plates of prosthodontic appliances. The chemical composition of the materials is shown in Table 2. Table 3 contains the technical specifications of tested materials according to the product cards. Samples were prepared in accordance with PN-68/C-89028 norm—a rectangular shape with dimensions of 15 mm × 10 mm × 3.5 mm. At a height of 5.5 mm from the base, there was an equilateral triangle-shaped notch with a rounded apex with a base of 0.8 mm, extending to 1/3 of the thickness of the sample. Samples for 3D printing were designed in Thinkercad (Autodesk, San Francisco, CA, USA). An Asiga Max UV 385 printer (Asiga, Alexandria NSW 2015, Australia) was used with a layer thickness of 0.05 mm. This printer has a DLP-LED 385 nm projector and layers of resin are cured using light. Cleaning of printed materials was made according to the manufacturer’s recommendations. NexDent Denture 3D+ was cleaned for 3 min in ethanol (>90%) in an ultrasonic bath to remove the excess resin, cleaned for 2 min in clear ethanol, and dried for 10 min. Dentona Optiprint Laviva was cleaned for 5 min in a non-heated ultrasonic bath in isopropanol (99%) with final cleaning made with detergent and water in a cold ultrasonic bath for 5 min. Saremco was cleaned with an alcohol-soaked (96%) cloth, brushed with alcohol solution, and dried with an air syringe. All 3D-printed materials were also polymerized in dedicated conditions. Samples made of conventional acrylic material were made by thermal polymerization technique (Figure 1). The mix ratio of Verted Dental Rapid Simplified was 1 mL to 2.3 g. The curing process was started at 100 °C, the water temperature was maintained for 20 min, and then it was air-cooled to ambient temperature.

Eleven samples from each group were subjected to three sterilization processes, and eleven were the control group. Pressurized steam sterilization at 121 °C, pressure 110 kPa, sterilization time 20 min in a SUN 18-II class B autoclave (SUN Medical, Ningbo, China), ethylene oxide sterilization at 55 °C for 60 min with 12 h aeration time (Steri-Vac GS5-2D, 3M, Saint Paul, MN, USA) and radiation sterilization (Elektronika 10/10 gas pedal, NPO Torij, Moscow, Russia), dose 25 kGy, electron energy 10 MeV. The autoclave sterilization usually has two standardized sterilization processes—in the temperature of 121 °C for 15–30 min (1 atm pressure) or 134 °C for 3–4 min (2 atm pressure). The program with lower temperature and lower pressure was chosen due to the lower risk of negative impact of high temperature and pressure on samples. For ethylene oxide sterilization, parameters were chosen by the technician to meet the criteria of sufficient sterility level—SAL (Sterility Assurance Level). The dosage of 25 kGy for radiation sterilization was used to reach SAL 6—the probability of not more than one viable microorganism in an amount of one million of sterilized items of the final product [9,11].

The impact strength of the samples was tested in a HIT 5.5P instrument (Zwick Roell, Ulm, Germany). Statistical analysis was performed using IBM SPSS Statistics 29.0.2 software. Due to the non-obtaining of a normal distribution in the Shapiro–Wilk tests, it was decided to perform non-parametric Kruskall–Wallis tests. Since more than two groups were compared, a multiple comparisons test was used to determine which groups were statistically significantly different.

## 3. Results

The tested materials are statistically significantly different in all variables when compared with samples which were not sterilized. The zero hypothesis was rejected as there were statistically significant differences between the tested groups. Three levels of statistical significance were applied (from highest to lowest: *p* < 0.001—marked in tables as ***, *p* < 0.01—marked **). Significantly higher energy and impact strength were recorded in Vertex than in all others, and significantly lower angle in Vertex than in all others (Table 4). For autoclave sterilization, statistically significantly higher energy and impact strength were found in Vertex than in Saremco and Dentona, and in Nextdent than in Dentona. In contrast, significantly greater angle was observed in Nextdent and Saremco than in Vertex, and in Nextdent than in Dentona (Table 5). 

The materials tested are statistically significantly different in all variables after ethylene oxide sterilization. There was significantly higher energy and impact strength in Vertex than in all others. A statistically significantly smaller angle was recorded in Vertex than in all other materials (Table 6).

After radiation sterilization, statistically significantly higher energy and impact strength were observed for Vertex than for all other materials. Vertex, on the other hand, had a statistically significantly lower angle than all other materials (Table 7).

The individual sterilization processes were then compared in terms of the variables analyzed separately for the different materials. In the Nextdent material, significantly higher energy appeared with ethylene oxide sterilization than with radiation or no sterilization, and with autoclave sterilization than with no sterilization. Significantly higher impact strength occurred in autoclave and ethylene oxide sterilization than in radiation sterilization. On the other hand, a significantly higher angle was recorded in radiation sterilization and in the absence of sterilization than in autoclave and ethylene oxide sterilization (Table 8). In the Saremco material, the Kruskal–Wallis test showed significantly higher energy and impact strength after ethylene oxide sterilization than in no sterilization or autoclave sterilization. In contrast, a significantly higher angle was recorded in no sterilization and autoclave sterilization than in ethylene oxide sterilization (Table 9).

In Dentona material, statistically significantly greater energy and impact strength were found in ethylene oxide and radiation sterilization than in autoclave sterilization, and in radiation sterilization than in no sterilization. In contrast, a significantly higher angle was found in autoclave sterilization and no sterilization than in radiation sterilization, and in autoclave sterilization than in ethylene oxide sterilization (Table 10).

The Vertex material showed no statistically significant differences between sterilization processes in any of the variables analyzed (Table 11).

## 4. Discussion

Surgical templates and removable prosthetic restorations that have contact with open wounds, such as immediate obturation plates, must be sterile. With the rapid advancement of material technology, many new polymers are being developed. Our study shows that 3D-printed resins for denture plates have lower values for flexural strength, modulus of elasticity, and fracture resistance than conventional acrylic material. This can be explained by the different reactivity of the printed resin’s monomers and curing conditions, as well as a lower double-bond conversion rate compared to conventional acrylic resins. In addition, weak bonding between successive printed layers may be the reason for the inferior mechanical properties. However, 3D-printing materials meet ISO requirements for strength, so they can be considered as an option when fabricating prosthesis restorations, considering the benefits of 3D-printing technology [6,33,34,35]. As the available resins for dental prosthesis plates have different molecular structures, they can be affected differently by the sterilization methods used.

The most widely used sterilization method is fast, non-toxic, and inexpensive steam sterilization under pressure. Four parameters of such sterilization are important: steam, pressure, temperature, and time. The high temperatures used (usually 121–132 °C) can destroy the structure of some polymers and adversely affect their properties [15,19]. Ethylene oxide sterilization is widely used in medicine because of its applicability to products that are not resistant to radiation and high temperatures. In addition, the process has been shown to be compatible with most polymers and does not lead to structural, property, and color changes in them [15,19,20]. Ionizing radiation affects the physical and chemical properties of polymers, but some plastics are resistant to degradation at the doses used for sterilization. The extent and type of changes in a particular polymer depend on its composition, the addition of stabilizers, the dose of irradiation used, and the time of irradiation. The presence of quaternary carbon atoms, halogen atoms (in halogenated polymers) and C-O-C bonds in the polymer chains, functional groups, degree of crystallinity, and the presence of oxygen, fillers and antioxidants are crucial to the radiation chemistry of polymers. While the absence of quaternary carbon atoms enhances cross-linking reactions, their presence promotes the cleavage of polymer chains and their degradation. In addition to chemical structures, the crystallinity/amorphousness ratio also plays an important role in the radiation effects of polymers. Highly amorphous materials are generally resistant to radiation due to their high ductility and elongation before fracture, and tolerance to many cleavages without disintegration [15,19,26,36]. After radiation sterilization, color changes in materials occur before measurable changes in physical properties [15]. Radiation sterilization of polymers can be used after careful analysis of possible effects [15,19].

The effects of various sterilization methods on polymethylmethacrylate and components made from it have been described in the literature. Autoclave sterilization is the simplest and most widely used method [7,16,18,37]. In a study by Münker et al. [8], sterilization of specimens made of Vertex Self-Curing (Vertex-Dental, Soesterberg, The Netherlands), Palacos R+G (Heraeus, Hanau, Germany) and NextDent C&B MFH (NextDent, Soesterberg, The Netherlands) containing PMMA in an autoclave caused deformation and exfoliation of the specimens due to high temperatures and steam pressure. The PMMA samples sterilized using pressurized steam sterilization were excluded by the authors from the results and further statistical analysis. In our study, we did not observe this phenomenon for Vertex Rapid Simplified (Vertex-Dental, Soesterberg, The Netherlands) material having polymethylmethacrylate in its composition. This study also showed no effect of ethylene oxide on the mechanical properties of PMMA, which is consistent with our results. Previous studies have shown that the action of low doses of ionizing radiation on PMMA components decreases their mechanical properties due to the cleavage of carbon chains, while the action of higher doses improves the mechanical properties of PMMA structures by increasing cross-linking [32,38]. Tatara et al. [28] studied the effect of electron beam sterilization on the mechanical properties of PMMA-based porous bone cement at doses of 30 kGy and 40 kGy. Their study showed a significant color change in the samples after radiation sterilization similarly to our study. In addition, they showed that 30 kGy and 40 kGy electron beam sterilization did not increase the cross-linking of the porous polymer but affected the polymerization of unbound monomer and small chains. The improved mechanical properties of porous PMMA may be caused by polymerization of the free monomer after electron beam sterilization, which is not observed in non-porous (solid) PMMA. Therefore, it was concluded that electron beam sterilization does not degrade porous PMMA and may even improve some mechanical properties. In the case of non-porous methyl methacrylate samples, as in our study, small amounts of unpolymerized monomer do not affect the strength parameters of the samples. The study by Behr et al. [12] also demonstrated a significant effect of the electron beam on the color change of acrylic polymer samples. In addition, they showed that the use of electrons with an acceleration energy of 10 MeV and a dose of 25 kGy results in improved fracture toughness, hardness of polymethylmethacrylate samples, and an increase in the energy required to fracture them, but the observed differences were small. In our study, we found no statistically significant differences in the energy and impact strength of PMMA samples after radiation sterilization. Similarly, Sharifi’s [11] study of medical-grade PMMA discs (Rod number 2, PolyOne; Littleton, MA, USA) showed no statistically significant differences in mechanical properties after 25 kGy electron beam treatment. These results suggest that electron beam sterilization at 25 kGy can be applied to PMMA components.

There is a lack of studies evaluating the effects of different sterilization methods on the mechanical properties and dimensions of removable prostheses made with 3D printing technology. The current evaluation is the first to compare the influence of different sterilization methods on mechanical properties of 3D-printed materials that may be used for denture bases of immediate obturators, as well as a whole range of different types of prosthodontic restorations. Monomers found in light-curing composite resins, such as urethane dimethacrylate (UDMA), are used in resins for 3D-printing models, surgical templates, and prosthetic restorations. However, due to a high molecular weight and high viscosity, they can interfere with the printing process. Therefore, non-hydroxylated monomers, such as ethoxylated bisphenol A-dimethacrylate (BisEMA), with lower viscosity are used. Additionally, triethylene glycol dimethacrylate (TEGDMA) can be added as a solvent to reduce the viscosity of the resin [39,40,41,42]. Yazigi et al. [43] evaluated the effect of steam sterilization under pressure at 121 °C on the dimensions of printed surgical templates. They showed that steam sterilization at 121 °C in an autoclave affects dimensional changes, although these changes are clinically acceptable. In addition, they showed that, depending on the 3D printing resin chosen, the resulting templates can have different dimensions even before sterilization. Török et al. [44] evaluated the effect of steam sterilization under pressure at 121 °C for 20 min on dimensional changes and mechanical properties of 3D-printed surgical guides. Statistical analysis did not detect any significant deformation compared to the control group, which was not sterilized. As for mechanical properties, no effect on flexural or compressive strength was detected. The results of Marei et al. [45] also showed no statistically significant effect of steam sterilization at 121 °C for 20 min on dimensional changes of printed surgical templates. A study by Pop et al. [46] examined the effect of steam sterilization at 121° C on the mechanical properties of 3D-printed samples and 3D-printed surgical templates for orthodontic mini-implants. They showed that DLP-printed specimens that underwent steam sterilization at 121° C showed a significant increase in flexural strength, flexural modulus, tensile strength, and tensile modulus compared to the control group. On the other hand, DLP 3D-printed surgical guides for orthodontic mini-implants subjected to steam sterilization at 121° showed a significant increase in maximum compressive load. Tangpothitham et al. [47] evaluated the effect of autoclave sterilization on the monomer release and mechanical properties of a 3D-printing resin for occlusal splints with high monomer content of UDMA, HEMA, and EGDMA. Their study showed that autoclave treatment after 3D resin polymerization reduced monomer release and improved surface microhardness without compromising bending modulus. The high temperatures achieved by autoclave treatment can reduce the residual monomer contents through its volatilization and additional polymerization, which also increases the microhardness of the samples. In addition, no significant changes in the dimensions of the samples were observed after autoclave sterilization at 121 °C. Marturello and Déjardin [48] studied the effect of different sterilization techniques (including steam and ethylene oxide) on the dimensional accuracy after sterilization of a standard surgical template 3D-printed with stereolithographic technology from biocompatible resins containing various methacrylate monomers by Formlabs (Formlabs, Somerville, MA, USA). Their results showed that the dimensional change of the evaluated biomaterials after sterilization was minimal. Ethylene oxide caused the largest dimensional changes in some materials, but the average dimensional changes after sterilization remained less than or equal to 0.05 mm. The dimensions of 3D-printed resin objects and their strength parameters are influenced by the printing technique and type of printer, the location of the supports and the orientation of the model on the platform, the chemical composition of the resins, the type and size of the printed objects, the final polymerization, and the evaluation methods [6,43,48,49]. The limitations of the presented study are that the research concentrated on mechanical properties of the evaluated materials without the verification of the sterility of the samples. Further studies are being prepared on this subject. The strong point of the research was providing clinically important recommendations on the type of sterilization process that could be applied to different 3D-printed denture base materials without deterioration of their durability.

## 5. Conclusions

Conventional acrylic material has higher impact strength than 3D-printing materials, and the sterilization methods used do not affect its strength parameters. The recommended methods of sterilization of prosthetic restorations made of 3D-printing materials in terms of impact strength influence are: Nextdent—in an autoclave or with ethylene oxide, Saremco—with ethylene oxide, and Dentona—with ethylene oxide or radiation.

## Figures and Tables

**Table 1:** Comparison of different sterilization methods that may be used for medical appliances.

Sterilization Method	Advantages	Disadvantages
Pressurized steam sterilization	excellent availability—all dental offices are equipped with autoclaves nontoxic to patient, staff, environment cycle is easy to control and monitor penetrates medical packaging, device lumens low costs well-tested influence on dental materials cycle is easy to control and monitor	cannot be used for materials fragile to heat, high pressure, or moisture potential for burns
Ethylene oxide sterilization (ETO)	method available for use in hospitals good penetration of complicated surfaces safe for heat-sensitive materials compatible with most materials used in medicine products can be processed in sealed, final packaging simple to operate and monitor best materials compatibility profile of all sterilization technologies	surface sterilization—does not sterilize the internal structure of the material requires aeration time to remove ETO residue ETO is toxic, a carcinogen, and flammable lengthy cycle/aeration times employee exposure ETO residuals may remain on device and package complex process
Radiation sterilization	sterilization of the internal structure of the materials safe for heat-sensitive materials fast process no residue on sterilized products compatible with most medical materials products can be processed in sealed, final packaging	high cost of equipment low availability ionizing radiation affects all polymers’ physical and chemical properties individual materials need to be assessed E-beam is not suitable for products with challenging product geometries and regions of high-density materials

**Table 2:** The chemical composition of tested materials.

Material	Manufacturer	Components
Denture 3D+	NextDent, Netherlands	Ethoxylated bisphenol A dimethacrylate (BisEMA) ≥ 75% 7,7,9 (or 7,9,9)-trimethyl-4,13-dioxo-3,14-dioxa-5,12-diazahexadecane-1,16-diyl bismethacrylate 10–20% 2-hydroxyethyl methacrylate (HEMA) 5–10% Silicon dioxide 5–10% Diphenyl(2,4,6-trimethylbenzoyl) phosphine oxide 1–5% Titanium dioxide < 0.1%
Denturetec	Saremco, Switzerland	Aliphatic urethane dimethacrylate (UDMA) 25–<50% Ethoxylated bisphenol-A dimethacrylate (BisEMA) 25–<50% Triethylene glycol dimethacrylate (TEGDMA) 1–<5%
Optiprint Laviva	Dentona, Germany	Aliphatic difunctional methacrylate < 40% Cristobalitmehl < 20% Aliphatic urethane acrylate < 10% 2,2′-ethylenedioxydiethyl dimethacrylate < 10% Siliziumdioxid < 6% 2-Propenoic acid < 5% Diphenyl(2,4,6 trimethylbenzoyl)phosphine oxide < 2%
Rapid Simplified	Vertex Dental, Netherlands	Methyl methacrylate; methyl 2-methylprop-2-enoate; methyl 2-methylpropenoate ≥ 75% Ethylene dimethacrylate < 10%

**Table 3:** The mechanical properties of tested materials according to product cards.

Material	Manufacturer	Specification
Denture 3D+	NextDent, Netherlands	Flexural strength—84 MPa, ISO 20795-1 Flexural modulus—2383 MPa
Denturetec	Saremco, Switzerland	Flexural strength >100 MPa, ISO 4049 Flexural modulus > 1000 MPa
Optiprint Laviva	Dentona, Germany	Flexural strength > 65, ISO 20795-1 Flexural modulus > 2000 MPa
Rapid Simplified	Vertex Dental, Netherlands	Flexural strength > 65, ISO 20795-1 Flexural modulus > 2000 MPa

**Table 4:** Comparison of materials without sterilization.

	Nextdent (1)			Saremco (2)			Dentona (3)			Vertex (4)			Kruskall–Wallis Test		R.I.S
	M	Me	SD	M	Me	SD	M	Me	SD	M	Me	SD	H	*p*	
energy (J)	0.007	0.007	0.001	0.007	0.007	0.001	0.007	0.007	0.001	0.029	0.025	0.010	27.131	<0.001 ***	4 > 1, 2, 3
impact strength (J/cm^2^)	0.029	0.028	0.004	0.031	0.029	0.003	0.029	0.028	0.003	0.112	0.104	0.033	29.048	<0.001 ***	4 > 1, 2, 3
angle [°]	86.43	86.40	0.22	86.38	86.40	0.22	86.49	86.58	0.16	80.44	81.54	2.85	25.824	<0.001 ***	1, 2, 3 > 4

**Table 5:** Comparison of materials after autoclave-pressurized steam sterilization.

	Nextdent (1)			Saremco (2)			Dentona (3)			Vertex (4)			Kruskall–Wallis Test		R.I.S
	M	Me	SD	M	Me	SD	M	Me	SD	M	Me	SD	H	*p*	
energy (J)	0.009	0.009	0.001	0.007	0.007	0.001	0.007	0.007	0.001	0.033	0.026	0.016	35.636	<0.001 ***	4 > 2, 3, 1 > 3
impact strength (J/cm^2^)	0.036	0.036	0.005	0.030	0.029	0.004	0.027	0.028	0.002	0.132	0.103	0.059	33.731	<0.001 ***	4 > 2, 3, 1 > 3
angle [°]	85.95	86.04	0.33	86.42	86.40	0.24	84.97	86.58	5.41	79.04	81.00	4.43	29.692	<0.001 ***	1, 2 > 4, 1 > 3

**Table 6:** Comparison of materials after ethylene oxide sterilization.

	Nextdent (1)			Saremco (2)			Dentona (3)			Vertex (4)			Kruskall–Wallis Test		R.I.S
	M	Me	SD	M	Me	SD	M	Me	SD	M	Me	SD	H	*p*	
energy (J)	0.009	0.009	0.002	0.009	0.008	0.001	0.008	0.008	0.001	0.036	0.030	0.013	29.973	<0.001 ***	4 > 1, 2, 3
impact strength (J/cm^2^)	0.038	0.036	0.006	0.035	0.034	0.003	0.032	0.033	0.004	0.141	0.126	0.044	28.283	<0.001 ***	4 > 1, 2, 3
angle [°]	85.87	85.95	0.41	86.07	86.13	0.19	86.27	86.31	0.21	78.23	80.10	3.65	29.223	<0.001 ***	1, 2, 3 > 4

**Table 7:** Comparison of materials after radiation sterilization.

	Nextdent (1)			Saremco (2)			Dentona (3)			Vertex (4)			Kruskall–Wallis Test		R.I.S
	M	Me	SD	M	Me	SD	M	Me	SD	M	Me	SD	H	*p*	
energy (J)	0.008	0.008	0.001	0.008	0.008	0.001	0.008	0.008	0.000	0.030	0.028	0.011	27.615	<0.001 ***	4 > 1, 2, 3
impact strength (J/cm^2^)	0.031	0.032	0.002	0.032	0.033	0.004	0.033	0.032	0.002	0.120	0.112	0.043	28.680	<0.001 ***	4 > 1, 2, 3
angle [°]	86.33	86.31	0.09	86.29	86.31	0.24	86.26	86.31	0.12	79.92	80.55	3.11	26.088	<0.001 ***	1, 2, 3 > 4

**Table 8:** Comparison of different sterilization methods of Nextdent material.

	Sterilization Method												Kruskall–Wallis Test		R.I.S
	Without (1)			Autoclave (2)			Ethylene Oxide(3)			Radiation (4)					
	M	Me	SD	M	Me	SD	M	Me	SD	M	Me	SD	H	*p*	
energy (J)	0.007	0.007	0.001	0.009	0.009	0.001	0.009	0.009	0.002	0.008	0.008	0.001	19.298	<0.001 ***	3 > 1, 4, 2 > 1
impact strength (J/cm^2^)	0.029	0.028	0.004	0.036	0.036	0.005	0.038	0.036	0.006	0.031	0.032	0.002	17.745	<0.001 ***	2, 3 > 4
angle [°]	86.43	86.40	0.22	85.95	86.04	0.33	85.87	85.95	0.41	86.33	86.31	0.09	21.257	<0.001 ***	1, 4 > 2, 3

**Table 9:** Comparison of different sterilization methods of Saremco material.

	Sterilization Method												Kruskall–Wallis Test		R.I.S
	Without (1)			Autoclave (2)			Ethylene Oxide(3)			Radiation (4)					
	M	Me	SD	M	Me	SD	M	Me	SD	M	Me	SD	H	*p*	
energy (J)	0.007	0.007	0.001	0.007	0.007	0.001	0.009	0.008	0.001	0.008	0.008	0.001	12.916	0.005 **	3 > 1, 2
impact strength (J/cm^2^)	0.031	0.029	0.003	0.030	0.029	0.004	0.035	0.034	0.003	0.032	0.033	0.004	14.018	0.003 **	3 > 1, 2
angle [°]	86.38	86.40	0.22	86.42	86.40	0.24	86.07	86.13	0.19	86.29	86.31	0.24	12.548	0.006 **	1, 2 > 3

**Table 10:** Comparison of different sterilization methods of Dentona material.

	Sterilization Method												Kruskall–Wallis Test		R.I.S
	Without (1)			Autoclave (2)			Ethylene Oxide(3)			Radiation (4)					
	M	Me	SD	M	Me	SD	M	Me	SD	M	Me	SD	H	*p*	
energy (J)	0.007	0.007	0.001	0.007	0.007	0.001	0.008	0.008	0.001	0.008	0.008	0.000	21.778	<0.001 ***	3, 4 > 2, 4 > 1
impact strength (J/cm^2^)	0.029	0.028	0.003	0.027	0.028	0.002	0.032	0.033	0.004	0.033	0.032	0.002	23.750	<0.001 ***	3, 4 > 2, 4 > 1
angle [°]	86.49	86.58	0.16	84.97	86.58	5.41	86.27	86.31	0.21	86.26	86.31	0.12	17.804	<0.001 ***	1, 2 > 4, 2 > 3

**Table 11:** Comparison of different sterilization methods of Vertex material.

	Sterilization Method												Kruskall–Wallis Test		R.I.S
	Without (1)			Autoclave (2)			Ethylene oxide(3)			Radiation (4)					
	M	Me	SD	M	Me	SD	M	Me	SD	M	Me	SD	H	*p*	
energy (J)	0.029	0.025	0.010	0.033	0.026	0.016	0.036	0.030	0.013	0.030	0.028	0.011	2.802	0.423	
impact strength (J/cm^2^)	0.112	0.104	0.033	0.132	0.103	0.059	0.141	0.126	0.044	0.120	0.112	0.043	2.819	0.420	
angle [°]	80.44	81.54	2.85	79.04	81.00	4.43	78.23	80.10	3.65	79.92	80.55	3.11	2.973	0.396	

## Data Availability

Data may be acquired from the authors of the study upon reasonable request.
